# Rupture of Multiple Splenic Artery Aneurysms: A Common Presentation of a Rare Disease with a Review of Literature

**DOI:** 10.4103/1319-3767.45061

**Published:** 2009-01

**Authors:** Ahmad Zubaidi

**Affiliations:** Department of Surgery, King Khalid University Hospital, Faculty of Medicine, King Saud University, Riyadh, Saudi Arabia

**Keywords:** Fibromuscular dysplasia, liver cirrhosis, portal hypertension, rupture, splenic artery aneurysm, sudden death

## Abstract

The splenic artery is the most frequent site of visceral arterial aneurysms. Usually a splenic artery aneurysm occurs as a single event; rupture is frequent, sometimes occurring as the first symptom and is sometimes fatal. This article presents a case of ruptured multiple splenic artery aneurysms—the symptoms and signs, operative and perioperative management, as well as a literature review of this clinically important entity.

Among visceral aneurysms, the splenic artery aneurysm (SAA) is the most common, representing 60% of such lesions.[1] There are 400 cases of SAA reported in the literature, 100 of which occur during pregnancy.[2] It is also the third most common site of intraabdominal aneurysms after the aorta and the iliac arteries. Although arterial aneurysms are more common in males, SAA occurs predominantly also in females,[3,4] being four times more common, and most of the women are pregnant when the lesion is first discovered. A splenic artery aneurysm is usually single and isolated and is ≤ 3 cm in size, whereas giant aneurysms (diameter ≥ 10 cm) are rare.[5,6] It is usually located in the mid or distal portion of the splenic artery, frequently at an arterial bifurcation. The exact prevalence is unknown as most SAAs are asymptomatic, although autopsy reports reveal a prevalence of 0.01–10.4%. They are found incidentally on 0.78% of angiograms[7–9] and in 7.1% of patients with cirrhotic portal hypertension.[10] Ruptured SAA is not often considered as a differential diagnosis of abdominal pain or, more importantly, in sudden collapse.

## CASE REPORT

A 42-year-old mother of four and 5/12 postpartum, presented to the King Fahad Medical City emergency room with a history of sudden onset of left-sided abdominal pain lightheadedness and repeated vomiting and hematemesis during the last two episodes. She was not on any medication or contraceptives. Her initial vital signs were stable, but a few minutes later, she became hypotensive (BP = 65/50 mm Hg), tachycardiac (HR = 110 bpm), and acidotic (pH = 7.1).

She was resuscitated and stabilized. The results of her initial investigations revealed normal plain X-rays. The complete blood count showed leucocytosis of 22,000 cells/dL3 and Hb = 6.8 g/dL. A spiral computed tomography (CT) of the chest and the abdomen revealed a 1.5-cm SAA about 1 cm from the origin of the splenic artery, a second 1 cm aneurysm in the middle of the artery, and a third larger aneurysm that measured about 4.5 cm at the hilum of the spleen [Figure 1]. No intravenous contrast leak was noted. The celiac axis and splenic arteriograms confirmed the CT-scan findings [Figure 2]. The distal of the two aneurysms were successfully embolized using both an absorbable gelatin sponge cube (Gelfoam) and an intraarterial coil. Because of the position of the proximal SAA, which was proximal to the celiac axis, and because of possible catastrophic complications, it was left alone as it was not the source of the intraabdominal bleeding. The patient remained hypotensive despite aggressive management and therefore, underwent surgery.

**Figure 1a:**
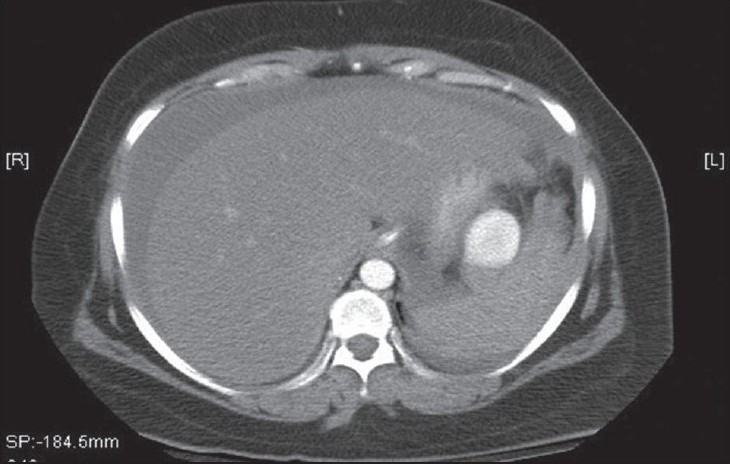
Spiral CT images with intravenous contrast enhancement

**Figure 1b:**
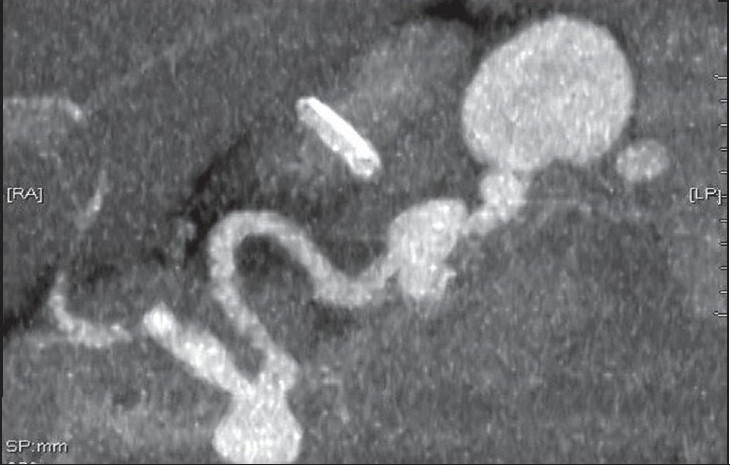
Spiral CT images with intravenous contrast enhancement

**Figure 1c:**
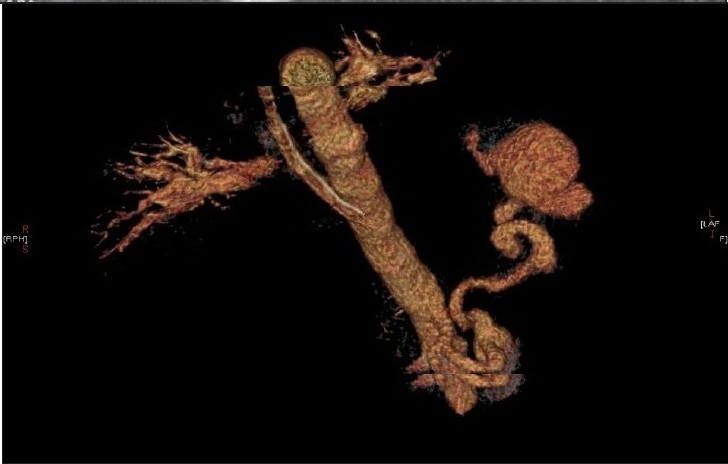
Reconstruction view of the spiral CT scan shows the splenic artery aneurysm. There is no evidence of calcification in the wall of the splenic aneurysm

**Figure 2a:**
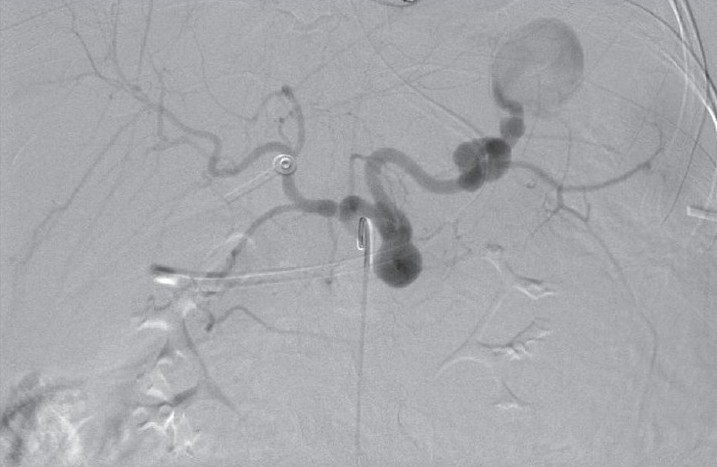
Early selective splenic artery arteriogram demonstrates three aneurysms

**Figure 2b:**
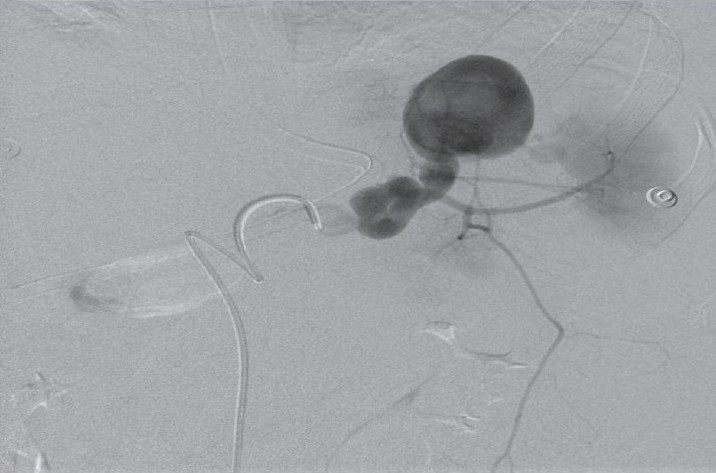
Late phase showing the most distal and the largest aneurysm containing thrombus

**Figure 2c:**
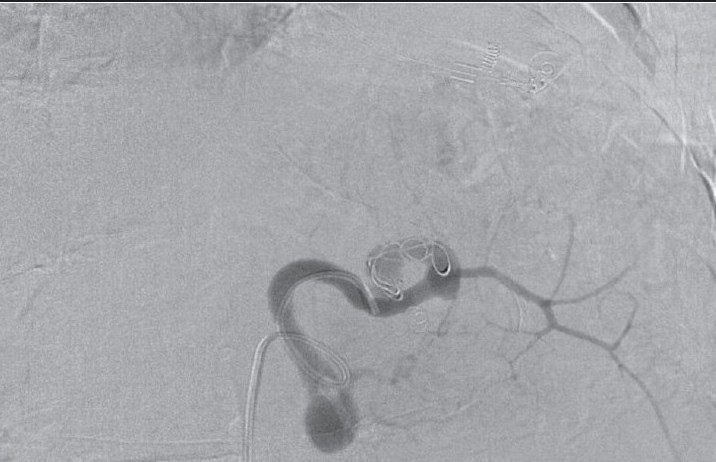
Postembolization splenic arteriogram shows coil occlusion of the mid and distal splenic artery, and nonfilling of the aneurysms with preservation of an early branch to the spleen

A midline laparotomy was performed and supraceliac aortic access was obtained. Proximal control was achieved by double ligation and by the division of the splenic artery close to its origin. A splenectomy was then performed, after which the patient had an unremarkable recovery and was discharged in a stable condition.

Pathological examination showed two intact proximal aneurysms and a ruptured distal aneurysm, the widest measuring 3.5 × 2.1 × 4.5 cm, with an intraluminal thrombus. Microscopic examination (chromatin stain) revealed a very thin wall of the aneurysms and a lack of elastin with a large infracted area. There was no evidence of vasculitis or neoplasm.

## DISCUSSION

Although the rupture of a splenic artery aneurysm is a rare cause of acute abdominal pain, it is a very important item in its list of the differential diagnosis, especially when it is associated with hypovolemic shock. This topic was identified by literature searches in Medline, Embase, and the Cochrane Library. The search string (free Text Terms and Medical Subject Headings [MeSH]) for rupture splenic artery aneurysm consisted of a combination of acute pain services, abdominal pregnancies, and splenic artery. The searches were limited to English language journals.

The splenic artery is the most frequent site of visceral arterial aneurysms.[1,11,12] The rupture of an SAA was first reported by Beaussier in 1770.[1] Generally, SAAs manifest in one of three ways: (1) as an incidental finding at angiography, laparotomy, or postmortem,[13] (2) as abdominal pain of varied severity, and (3) as a rupture with hypotension and a sudden collapse in 2–10% of the cases.[14,15] Initially, the rupture may be tamponaded in the lesser omental sac with apparent clinical stabilization. Eventually, the blood escapes into the free peritoneal cavity, either through the foramen of Winslow or through rupture of the pars flaccida. This patient presented initially with left-sided abdominal pain associated with nausea, vomiting, and dizziness. Eight hours later, she developed hypotension and went into hypovolemic shock. This could be explained by the “double rupture” phenomenon which has been reported in 20–30% of ruptured splenic artery aneurysms.[1,16] Very few patients (5%) present with symptoms before the rupture of their SAA.[17,18] Other complications have been described such as ruptures into a hollow viscus or the pancreatic duct, and development of splenic arteriovenous fistulas. The overall mortality rate of ruptured splenic artery aneurysms is 10–25%.[13,16]

The cause of an SAA is unknown. However, local failure of the connective tissue of the arterial wall to maintain the integrity of the vessel could be playing a major role in the development of this phenomenon. This leads to fragmentation of elastic fibers and a loss of smooth muscle.[19] It is believed that there is a strong association between pregnancy and splenic artery aneurysm formation, especially in multiple pregnancies.[19,20] This possibly can happen through two mechanisms. (i) Hormonal effects of estrogen and progesterone whose receptor sites have been demonstrated in the arteries.[20] During pregnancy, the significantly elevated levels of these hormones can presumably lead to significant pathological alteration of the structure of the arterial wall. Furthermore, there is fibrodysplasia of the media with failure of elastin formation.[13,21] The result of this process is subsequent mural outpouching especially at bifurcations. (ii) The other mechanism is due to the effects of the physiological changes of increased blood volume and cardiac output induced by pregnancy. The effects are cumulative with each successive pregnancy such that not only the incidence of SAA but the frequency of rupture is also believed to increase as parity rises.[21] Another proposed factor is the hormone relaxin. This is secreted in late pregnancy and is thought to affect the elasticity of the splenic artery.[16] Other potential risk factors include portal hypertension, inherited vascular and connective tissue disorders, congenital abnormalities of the vessels, vascular trauma, inflammatory processes, and degenerative arterial disease. However, arteriosclerosis is rarely a primary causative factor.[10,13,21]

Arteriography is the gold standard for diagnosis in suspected unruptured aneurysms.[22,23] However, angiography still remains the most valuable investigative modality to precisely localize the source of bleeding and assess the collateral flow.[7] Ultrasonography with pulsed Doppler[24] and CT scans are also useful.[10,25] In cases of ruptured SAA present with acute abdominal pain, an emergency CT scan may reveal free fluid in the upper abdomen and may show the aneurysm and leaking of the intravenous contrast if bleeding is in persistent.[2,25–27]

Treatment of splenic artery aneurysms is recommended in patients with symptomatic or expanding aneurysms, patients with aneurysms > 2 cm, women of childbearing age, and liver transplantation candidates.[10,14,15,21] Several treatment options can be applied: aneurysm exclusion, excision, revascularization, and endovascular techniques. Historically, open surgical excision of the aneurysm, with or without splenectomy, is the conventional treatment which has well documented efficiency and durability.[28–30] Laparoscopic ligation has also been recently described with good results, but experience with this procedure is limited.[31,32]

In patients with hilar aneurysms such as the one presented here, coil/Gelfoam embolization is a viable option. If selective catheterization of the aneurysm cannot be performed, embolization of the entire splenic artery is an alternative option. Patients should be alerted to the potential for severe pain from splenic infarction. Our patient underwent embolization of the distal ruptured aneurysm to stabilize her hemodynamics. Unfortunately, she continued to be hypotensive despite aggressive resuscitation. She underwent an open excision of the splenic artery with splenectomy.

Endovascular treatment is an emerging therapy for SAA and other visceral aneurysms with constantly improving results. These minimally invasive endoluminal techniques may offer a distinct advantage to conventional repair. However, because of the relative paucity of visceral artery aneurysms, long-term results are yet to be reported. It offers the potential benefit of maintaining splenic perfusion while excluding the aneurysm, thereby eliminating the risk of rupture or infarction.[33,34] The natural history of true splenic artery aneurysms involves the elongation and increasing tortuosity of the vessel. Subsequently, stent delivery to a mid-splenic or distal-splenic artery aneurysm may be technically unattainable.[35] As such, endovascular stent grafting of these lesions may be confined to the proximal splenic artery. Further assessment of this mode of therapy is warranted for the treatment of splenic artery aneurysms. This mode of therapy could not be delivered to this patient because of instability of this patient as well as the presence of three aneurysms in the same vessel; one of which has already ruptured.

## CONCLUSION

A diagnosis of ruptured SAA should be considered in any woman who presents with severe left upper abdominal pain or hypovolemic shock. Successful treatment of SAA requires a high index of suspicion, early recognition, and prompt management. Treatment usually is challenging. However, safe surgical treatment can be applied by a carefully planned approach based on adequate exposure and proximal and distal arterial control.
